# CTL epitope distribution patterns in the Gag and Nef proteins of HIV-1 from subtype A infected subjects in Kenya: Use of multiple peptide sets increases the detectable breadth of the CTL response

**DOI:** 10.1186/1471-2172-7-8

**Published:** 2006-04-18

**Authors:** Jeffrey R Currier, Unchalee Visawapoka, Sodsai Tovanabutra, Carl J Mason, Deborah L Birx, Francine E McCutchan, Josephine H Cox

**Affiliations:** 1 The US Military HIV Research Program, Suite 200, 13 Taft Court, Rockville, MD 20850, USA; 2 Department of Enteric Diseases, Armed Forces Research Institute of Medical Sciences, 315/6 Rajvithi Road, Bangkok 10400, Thailand

## Abstract

**Background:**

Subtype A is a major strain in the HIV-1 pandemic in eastern Europe, central Asia and in certain regions of east Africa, notably in rural Kenya. While considerable effort has been focused upon mapping and defining immunodominant CTL epitopes in HIV-1 subtype B and subtype C infections, few epitope mapping studies have focused upon subtype A.

**Results:**

We have used the IFN-γ ELIspot assay and overlapping peptide pools to show that the pattern of CTL recognition of the Gag and Nef proteins in subtype A infection is similar to that seen in subtypes B and C. The p17 and p24 proteins of Gag and the central conserved region of Nef were targeted by CTL from HIV-1-infected Kenyans. Several epitope/HLA associations commonly seen in subtype B and C infection were also observed in subtype A infections. Notably, an immunodominant HLA-C restricted epitope (Gag 296–304; YL9) was observed, with 8/9 HLA-C_W_0304 subjects responding to this epitope. Screening the cohort with peptide sets representing subtypes A, C and D (the three most prevalent HIV-1 subtypes in east Africa), revealed that peptide sets based upon an homologous subtype (either isolate or consensus) only marginally improved the capacity to detect CTL responses. While the different peptide sets detected a similar number of responses (particularly in the Gag protein), each set was capable of detecting unique responses not identified with the other peptide sets.

**Conclusion:**

Hence, screening with multiple peptide sets representing different sequences, and by extension different epitope variants, can increase the detectable breadth of the HIV-1-specific CTL response. Interpreting the true extent of cross-reactivity may be hampered by the use of 15-mer peptides at a single concentration and a lack of knowledge of the sequence that primed any given CTL response. Therefore, reagent choice and knowledge of the exact sequences that prime CTL responses will be important factors in experimentally defining cross-reactive CTL responses and their role in HIV-1 disease pathogenesis and validating vaccines aimed at generating broadly cross-reactive CTL responses.

## Background

The development of an efficacious prophylactic vaccine for human immunodeficiency virus type 1 (HIV-1) is the goal of a concerted worldwide research effort [1,2]. Although the precise correlates of protective immunity against HIV-1 infection are not clearly defined, a large body of accumulated data suggests that an ideal HIV-1 vaccine will need to stimulate both humoral and cellular immune responses against the virus [3]. While chronic untreated HIV-1 infection causes a profound immunodeficiency, the initial HIV-1 infection stimulates strong cellular and humoral immune responses against the virus [4,5]. CD8 cytotoxic T lymphocytes (CTL) constitute a major component of the cellular arm of the immune response, and have a central role in the control of initial viremia immediately following HIV-1 infection and in the establishment of long-term AIDS free survival [6,7]. The ability to rapidly and accurately characterize CTL responses in HIV-1 infected individuals has grown exponentially in recent years. New technologies to detect CTL responses by measuring interferon-gamma (IFNγ) release, such as the enzyme linked immunospot (ELIspot) assay and cytokine flow cytometry (CFC), have been coupled with overlapping pooled peptide technology (OLP) to give detailed and precise analyses of HIV-1-specific cellular immune responses [8-11]. The fine mapping of T cell epitopes and the identification of immunodominant regions of HIV-1 gene products is integral to vaccine design, to the development of immunotherapeutic strategies, and to the optimization of assays for assessing vaccine efficacy.

Genetic diversity is the foremost obstacle for any screening method for HIV-1-specific cellular immunity [12-14]. Geographically defined epidemics can be characterized by the dominance of distinct genetic subtypes of HIV-1, with at least 9 subtypes and 21 CRFs of HIV-1 currently recognized [15]. Since intra-subtype amino acid sequence variation can be as high as 10–15% (depending on the viral gene product) and inter-subtype variation can be much higher, any single sequence of HIV-1 used for screening for CTL responses will differ considerably from the sequence of the infecting virus in an individual. This is an important complicating factor for studying the CTL response, because CTL are primed *in vivo *in response to the autologous infecting virus. Although the use of autologous viral sequences has been employed in several studies, their general use is impractical and labor intensive since, not only does the viral sequence between individuals differ significantly, but the viral quasi-species in a single individual can also take many different forms [16-18]. A proposed solution to this seemingly intractable problem has been to use OLP sets based upon computationally derived consensus or ancestral sequences [19,20]. Viral consensus and ancestral sequences have the theoretical advantage of being more related on average to any given *in vivo *viral sequence than any arbitrary viral isolate would be. A further complicating factor for OLP screening is that practical peptide libraries usually comprise 15–20-mers overlapping by 10–12 amino acids, but the optimal length of peptides that bind to MHC class I molecules is 8–11 amino acids. However, this problem is overcome in most cases by exogenous addition of the peptides at biologically excess concentrations [21-23]. Despite these compromises the utility of screening for CTL responses with consensus sequence based OLP has been demonstrated in several recent studies, which have comprehensively analyzed the full breadth and magnitude of cellular immune responses to the entire HIV-1 proteome [10,11,24-26]. Although consensus sequence based OLP have now become the *de facto *standard for studies of HIV-1 cellular immunity, the assumption that a subtype based consensus is implicitly better than a randomly chosen isolate from the same subtype and isolates from other subtypes has only recently been questioned formally [27-30].

This study was undertaken to assess the CTL response to the Gag and Nef proteins of HIV-1 infected individuals in Kenya, a predominantly HIV-1 subtype A endemic region, and to compare and contrast the CTL response measured with that determined by prior studies in subtype B and C epidemics. CTL responses directed against the *gag *and *nef *gene products were selected because previous studies have shown that these two gene products contain the highest epitope density and are the most frequently recognized HIV-1 proteins in subtype B and C infection [10,11,24-26,31]. Detection of Gag-specific CTL responses was performed using peptide libraries representing a subtype A consensus, a subtype A isolate, a subtype C isolate and a subtype D isolate, to gain an insight into subtype-specificity of CTL responses in a region of multiple subtype endemicity. Detection of Nef-specific CTL responses was performed using peptide libraries representing a subtype A isolate, a subtype C isolate and a subtype D isolate. After sequence analysis of the *gag *and *nef *genes from proviral DNA confirmed that the cohort was predominantly HIV-1 subtype A infected, we integrated knowledge of the sequence of the infecting viral isolate, the HLA-type of the subject and the CTL response made by that subject to: (1) determine the frequency of recognition and pattern of CTL immunodominance within the Gag and Nef proteins in subtype A infections; (2) compare the frequently recognized epitopes in subtype A infection with those previously determined for subtype B and C infections; (3) compare subtype matched and mismatched peptide based peptides sets for their ability to detect CTL responses; (4) characterize an immunodominant *HLA-C*_W_*03 *restricted CTL epitope from HIV-1. Within the framework of this study we were able to directly test the hypothesis that in a given cohort of HIV-1-infected subjects consensus and isolate-based peptide libraries derived from the homologous subtype would detect significantly broader and greater magnitude CTL responses than heterologous subtype-derived peptide libraries.

## Results

### HIV-1 *gag *and *nef *region subtype analysis and HLA-typing

**Figure 1:**
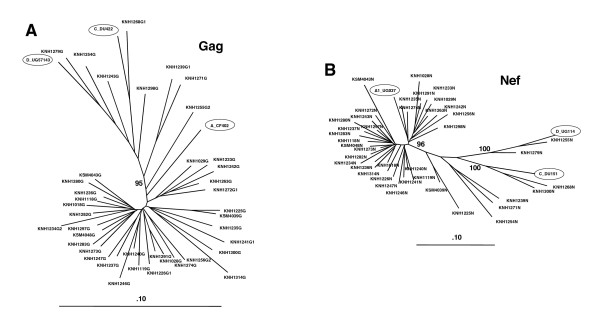
Phylogenetic trees of the *gag *(**A**) and *nef *(**B**) proviral DNA sequences obtained from the cohort. Circled are the DNA sequences of the isolates from which the screening peptide sets were deduced. The large cluster of sequences in both trees represents subtype A. Bootstrap values are shown.

Full-length *gag *and *nef *sequence analysis of PBMC proviral DNA was performed to characterize the HIV-1 subtype distribution within the cohort. A single full-length *gag *and *nef *sequence could be obtained from 40 of the 42 subjects (sequence could not be obtained from 2 subjects). In the *gag *region, 31 of the 40 subjects harbored HIV-1 subtype A (29 sub-subtype A1 and 3 sub-subtype A2), 1 had subtype D. The remaining 8 *gag *sequences represented recombinant structures, 5 A1/D, 3 A2/D. Within the *nef *region, 35 of 40 subjects harbored HIV-1 subtype A (32 sub-subtype A1 and 3 sub-subtype A2), while the remaining 5 sequences represented 2 subtype D, 2 subtype C and 1 A1/D recombinant. Figure 1 shows phylogenetic trees of the *gag *(A) and *nef *(B) sequences obtained from the cohort, with the DNA sequence from which the isolate based sequences were derived shown for clarity. For both regions, the subtype A isolate shows closer branching to the majority of the cohort sequences than the subtype C or subtype D isolates. A pair-wise comparison of the translated autologous virus protein sequences (from all 40 subjects) with the sequence of each of the peptide sets to be used for the ELIspot screening was performed. The autologous Gag protein sequences have mean differences of 6.1%, 10.2%, 11.6% and 13.3% from the subtype A consensus, the subtype A isolate, subtype C isolate and subtype D isolate based Gag peptide sets respectively. The autologous Nef protein sequences have mean differences of 16.4%, 18.8% and 24.5% from the subtype A isolate, subtype C isolate and subtype D isolate based Nef peptide sets respectively. Therefore, the cohort is predominantly infected with HIV-1 subtype A viruses, and as would be expected, the Gag subtype A consensus and Nef subtype A isolate based peptide sets are closest in sequence to the viruses present in the cohort. The nucleotide sequences derived in this study were submitted to GenBank and are available under the following accession numbers: [DQ367261–DQ367332, AY945736–AY945739].

All 42 subjects were high resolution HLA class I genotyped to 4-digit accuracy. The frequencies of the 10 most common HLA-A alleles were: A6802 (28.6%); A0201 (21.4%); A0101 (19.0%); A3601 (14.3%); A7401 (14.3%); A3001 (11.9%); A3002 (9.5%); A2902 (9.5%); A2301 (9.5%) A6601 (7.1%) and the 10 most common HLA-B alleles were: B5301 (28.6%); B1503 (16.7%); B4202 (14.3%); B4501 (11.9%); B1510 (9.5%); B0801 (9.5%); B4901 (9.5%); B5703 (9.5%); B5101 (7.1%); B8101; and the 10 most common HLA-C alleles were: C_W_0401 (31.0%); C_W_0701 (26.2%); C_W_0304 (21.4%); C_W_0602 (21.4%); C_W_17(01–03) (21.4%); C_W_16(01,02) (16.7%); C_W_0202 (14.3%); C_W_18(01,02) (9.5%); C_W_0802 (7.1%); C_W_1502 (7.1%). The detected allele frequencies are in close agreement with published HLA allele frequencies for Kenya [32,33] and hence, the cohort can be considered representative of the Kenyan population.

### Elispot Screening with Overlapping Peptide Pools

**Figure 2:**
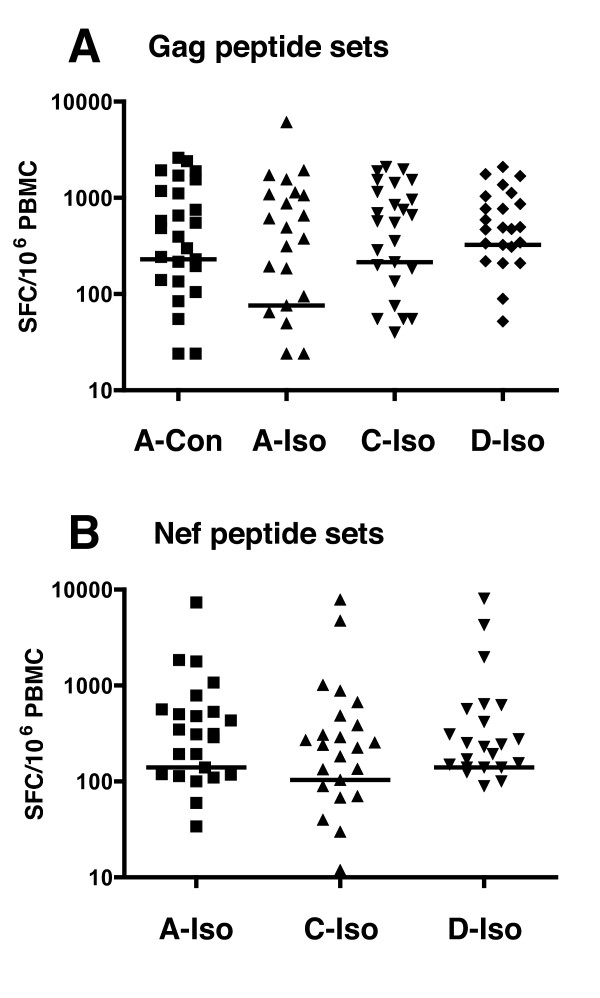
Magnitude of CTL responses measured for each subject in response to either Gag (**A**) or Nef (**B**) peptide sets (Con = consensus; Iso = isolate). For each study subject all detected ELIspot responses against each peptide set were totaled (expressed as SFC/10^6 ^PBMC) and plotted beside each other.

The cohort was screened for IFNγ T cell responses against four sets of Gag overlapping peptide pools and three sets of Nef overlapping peptide pools. The *gag *and *nef *genes were selected because these two gene products have been documented to contain the highest CD8 epitope density and are the most frequently recognized HIV-1 gene products [10,11,24-26]. As described in the Methods section, the sequences of the peptide sets used for the study were based upon homologous and heterologous subtype isolates, and on a computationally derived subtype A consensus sequence for the Gag protein. IFNγ ELIspot responses from cryopreserved PBMC were assessed by peptide matrix screening, followed by individual peptide confirmation assays in order to maximize the information from individual specimens. In both the screening and confirmation assays, positive responses were defined as those where the test wells exceeded the 99% confidence interval of replicates of six negative control wells, while for the confirmation assays individual peptides were tested in triplicate. The 42 subjects included in the study all demonstrated strong responses to the SEB positive control (range = 515 – 7795 SFU/10^6 ^PBMC, or confluence). Table 1 summarizes the IFNγ ELIspot screening data in terms of the number of subjects responding to each peptide set and the number of discrete responses detected. An epitope, or discrete epitopic region, was defined by a response to a single peptide or adjacent pair of overlapping peptides from an individual peptide set. While 33 subjects responded to at least 1 peptide from the 7 sets of screening peptides, 9 subjects showed no detectable Gag or Nef peptide specific response. Of the 33 subjects responding to any peptide set (Gag or Nef) 30 recognized at least 1 Gag peptide set. Of interest, similar numbers of subjects recognized at least 1 peptide from the 4 Gag peptide sets: the subtype A consensus was recognized by 26 subjects; the CRF01_AE peptide set by 25 subjects; the subtype C peptide set by 25 subjects; and the subtype D peptide set by 23 subjects. The total number of responses detected in all subjects was also evaluated. Among the Gag responding subjects a total of 65 responses were detected (mean of 2.2 epitopes per responding subject), with between 1 and 5 epitopes recognized. A slightly greater, although non-statistically significant, number of responses was detected by the subtype A consensus based peptide set compared with the isolate based peptide sets (χ^2 ^test). The subtype A Gag consensus and subtype A Gag isolate-based peptide sets detected 50 and 39 of the 65 total Gag responses respectively, while the subtype C and D peptide sets each detected 41 responses. The mean number of responses detected per responding subject was not notably different between the 4 Gag peptide sets (Table 1). The total frequencies of cells, expressed as SFC/10^6 ^PBMC, responding to each of the Gag peptide sets is shown in Figure 2A. Within the cohort the measured frequency of responding cells was similar for each peptide set and no statistical difference among the peptide sets was observed (Friedman Test and Kruskal-Wallis Test). Therefore, the ability to detect Gag derived CTL epitopes in an ELIspot assay for IFNγ production was not significantly improved by using a set of peptides based upon the consensus of subtype A compared with randomly chosen homologous and heterologous subtype based isolate sequences.

**Table 1:** Summary of responses detected against each of the peptide sets.

Peptide Set	Number of Responders	Number of Responses Detected	Epitopes per Responder^1^
Any Gag	30	65	2.2
Subtype A Gag Consensus	26	50	1.9
Subtype A Gag Isolate (90CF402)	25	39	1.6
Subtype C Gag Isolate (DU422)	25	41	1.6

Subtype D Gag Isolate (98UG57143)	23	41	1.8
Any Nef	24	40	1.7
Subtype A Nef Isolate (92UG037)	24	38	1.6
Subtype C Nef Isolate (DU151)	22	29	1.3
Subtype D Nef Isolate (94UG114)	22	32	1.5

^1^An epitope (or discrete epitopic region) was defined as a response to a single peptide or pair of adjacent peptides.

Of the 33 subjects responding to any peptide set, 24 recognized at least 1 Nef peptide set. The 3 Nef peptide sets were also recognized by equivalent numbers of subjects: the subtype A peptide set was recognized by 24 subjects; the subtype C peptide set by 22 subjects; and the subtype D peptide set by 22 subjects. A total of 40 Nef directed responses were detected among the Nef responders with between 1 and 3 Nef epitopes recognized by each subject (mean of 1.7 epitopes per responding subject). The subtype A Nef peptide set was significantly (χ^2 ^test; p < 0.03) better at detecting the Nef-specific responses (detecting 38 of the 40 total responses) compared with the heterologous subtype based peptide sets (subtype C, 29 responses; and subtype D, 32 responses). There was however, no statistically significant difference between the magnitude of SFC detected in response to each of the 3 Nef peptide sets (Figure 2B) (Friedman Test and Kruskal-Wallis Test).

### Multi-subtype reactive and subtype-specific CTL are detectable in HIV-1 subtype A infection

**Figure 3:**
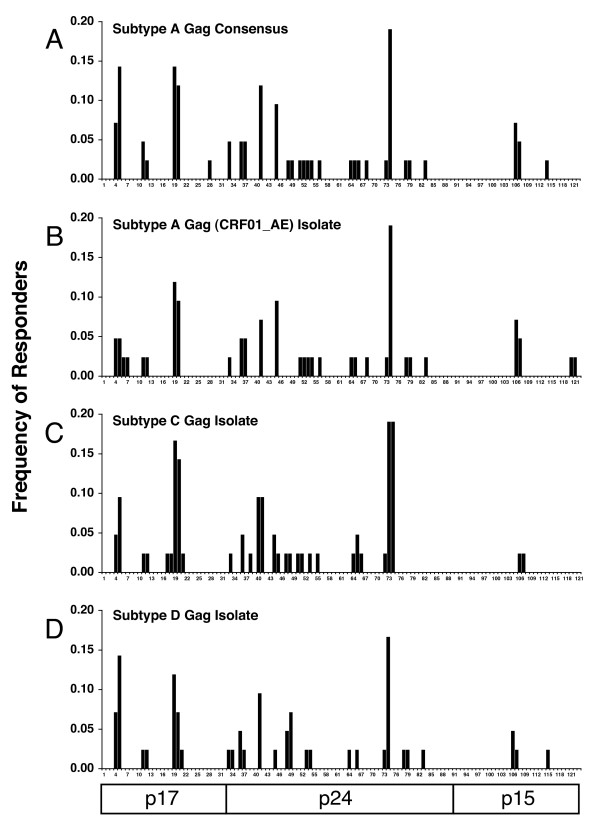
Frequency of response histograms for all peptides from each Gag peptide set. All peptide responses scored as positive (by cut-off criteria described in Methods) are shown, including those obtained for adjacent overlapping peptides. Histograms are shown for (**A**) consensus subtype A, (**B**) subtype A (CRF01_AE) isolate, (**C**) subtype C isolate, and (**D**) subtype D isolate. Peptides are numbered sequentially along the x-axis.

**Figure 4:**
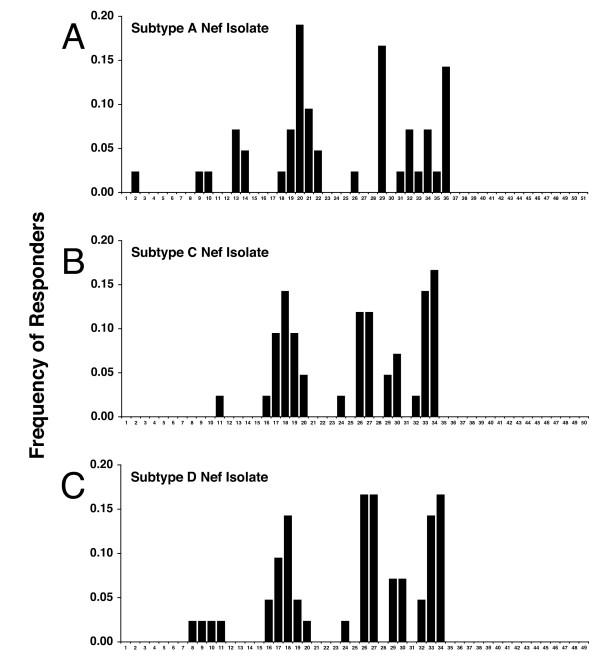
Frequency of response histograms for all peptides from each Nef peptide set. All peptide responses scored as positive (by cut-off criteria described in Methods and Materials) are shown, including those obtained for adjacent overlapping peptides. Histograms are shown for (**A**) subtype A isolate, (**B**) subtype C isolate, and (**C**) subtype D isolate. Peptides are numbered sequentially along the x-axis.

Figure 3 shows the cumulative frequency of responses against individual Gag peptides for the cohort. The profiles show a remarkably similar response pattern for each of the four Gag peptide sets, indicating a potentially high level of multi-subtype reactivity of Gag-specific CTL from subtype A infected subjects. Responses were directed primarily against the p17 (23 responses) and p24 (36 responses) proteins, with fewer responses detected against the p15 proteins (6 responses). Four epitopic regions of the subtype A Gag consensus peptide set were recognized by >10% of the subjects and were also cross-recognized within the isolate based peptide sets. Two of these regions were located in p17, peptides 4–5 and peptides 19–20 (amino acids 13–31 and 73–91 respectively) and two within p24, peptides 40–41 and peptides 73–74 (amino acids 161–175 and 289–307 respectively). These regions are rich in previously mapped CTL epitopes from subtypes B and C [15]. The study also showed a notable similarity in the response profiles against the 3 sets of Nef peptides (Figure 4). The central conserved region of the Nef protein is recognized predominantly in all 3 peptide sets, and is also extremely conserved among the peptide sets. From amino acid position 60 through to 148 (peptides 19–36 for subtype A, and peptides 17–34 for subtypes C and D) the three peptide sets have only 13 amino acids which differ, and a single amino acid insertion the subtype C isolate with respect to the subtype A and D isolates. Not surprisingly, the majority (32/40) of the Nef responses detected by the 3 peptide sets reside within this 89 amino acid stretch. The subtype A peptide set is slightly offset with respect the subtype C and D peptide sets due to an 11 amino acid insertion after residue 27. This insertion did not contain any detected epitopes, nor did it appear to disrupt epitopes detected with the subtype C and D peptide sets or other previously defined epitopes [15].

**Figure 5:**
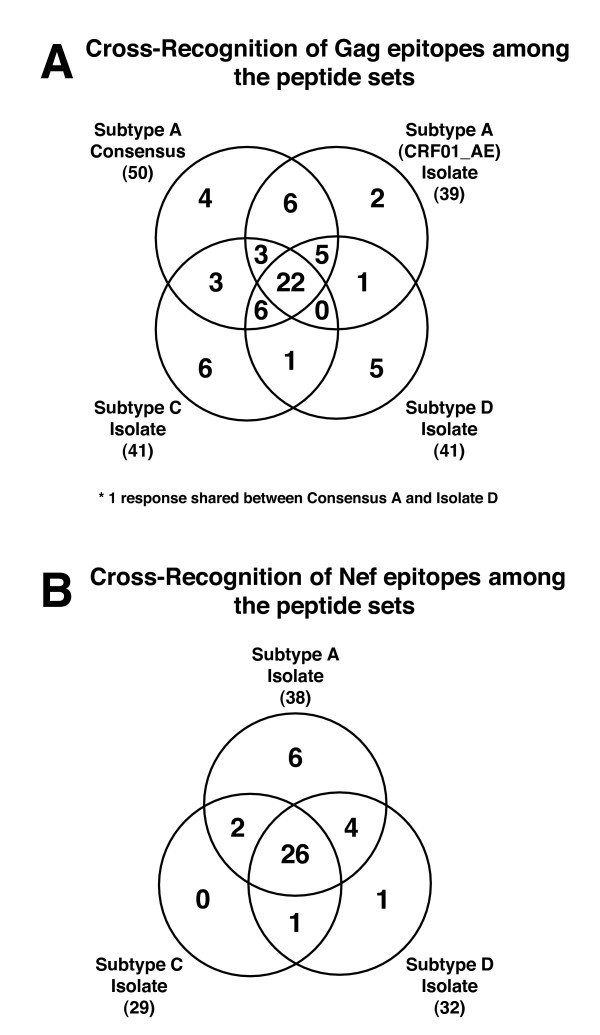
Venn diagram display of responses (epitopes) detected with either Gag (**A**) or Nef (**B**) peptide sets. Each overlapping region is annotated with the number of responses detected for that particular relationship. For example, the central region in panel A, which is bounded by all four circles, shows that 22 responses were detected by all four Gag peptide sets.

Since the overall analysis showed a high degree of similarity and multi-subtype recognition, the cohort was examined for the number of responses detected across all subtypes and those detected by a subset of subtypes or a single subtype. Overlapping Venn diagrams (Figure 5) were used to display the number of responses detected by the individual peptide sets and by the various combinations of peptide sets. For example, Figure 5A shows that of 65 identified Gag responses, 22 were detected with all 4 peptide sets as shown by the central overlap of all 4 circles, each of which encompasses all responses detected by that peptide set. By this analysis 36 of the 65 Gag responses were detectable with at least 3 of the peptide sets, while the remaining 29 were detected with only 2 or 1 of the peptide sets. Strikingly, the consensus subtype A peptide set detected only 4 responses not detectable by any of the 3 isolate based peptide sets, and the isolate based peptide sets collectively, detected 15 responses not detected with the subtype A consensus peptide set. Moreover, the subtype C and D heterologous isolate derived peptide sets detected 12 responses not detected by either of the two subtype A based peptide sets, with 11 of the responses representing different epitopic regions since only 2 were against the same peptide. It was reasoned that because the cohort contained 1 subtype C, 2 subtype D and 6 inter-subtype recombinant *gag *genes, then these subjects may be the source of the responses not detected with the subtype A derived peptide sets. However, this was not the case, as only 1 of the 12 epitopes not detected with subtype A peptide sets could be attributed to subjects from which non-pure subtype A *gag *containing virus sequence was obtained. This response was detected with the subtype C peptide set and was from a subject (KSM4039) carrying an inter-subtype recombinant *gag *sequence (A1/D). Thus, the overall magnitude and breadth of the CTL response against subtype A Gag was underestimated when screening with any single peptide set. The use of a single peptide set derived from an homologous subtype Gag sequence (whether it be based upon an isolate or a consensus) did not give a complete representation of the repertoire of Gag-specific epitopes recognized by any given subject in this study. Similar analysis for the Nef protein showed that the homologous subtype A derived peptide set detected all but 2 of the Nef responses detected in this study. Most of the Nef responses were directed to the highly conserved central region of the protein with 26/40 total responses detected by all 3 isolate derived peptide sets. Notably, the number of responses detected with the subtype A Nef isolate peptide set (38) was significantly greater than that detected with either the subtype C Nef (29) or D Nef (32) peptide sets (p = 0.03; χ^2 ^test). Therefore in this cohort, the use of homologous subtype derived Nef sequences did maximize the detection of Nef CTL epitopes.

### Characteristics of positive and discordant peptides in relation to each other and to autologous virus sequences

The identified CTL responses were next compared and examined in greater detail. All positively recognized peptides from all peptide sets were compared to the Gag or Nef sequence of autologous virus. First, the total number of possible responses detected in the study was calculated by multiplying the number of discrete Gag responses by 4 and the number of discrete Nef responses by 3. This calculation was performed since any response directed against Gag had 4 possible hits (one from each peptide set), and any response against Nef had 3 possible hits. Of a total of 260 possible responses directed against all Gag peptide sets, 171 were detected, while of a possible 120 responses against all Nef peptide sets, 99 were detected. When the peptides representing the 270 detected Gag and Nef responses were compared to the autologous virus sequence obtained from each host, approximately one-third (98) were identical to the autologous virus sequence while approximately two-thirds (172) had at least one amino acid mismatch with the autologous sequence. Hence a greater number of responses were detected in subjects whose proviral DNA contained a different sequence compared to the peptide used to detect the response. To obtain a better impression of the cross-reactive nature of the CTL responses measured, the impact of "frame-of-epitope" and sequence variation between concordant and discordantly recognized peptides was also examined. The "frame-of-epitope" refers to the offset position of a 9-mer minimal epitope within a given 15-mer screening peptide and the effect has been the subject of several recent studies [21,23,30]. Because the screening peptide sets were derived from quite disparate subtypes, there was significant frame offsetting between peptides mapping to the same region of the different peptide sets. When a given epitope was detected with more than one peptide set, there were 70 examples of frame offsetting between the concordant (positive) peptides (Table 2). This indicates that despite significant minimal epitope offsetting, cross-recognition of the peptide sets was frequent. In addition, within the concordant peptides there were 74 examples of peptides with different sequences able to be recognized. These most likely represent truly cross-reactive responses, since more than one peptide sequence can be recognized by CTL from a single individual.

**Table 2:** Comparison of concordant and discordant peptide responses.

	Concordant Peptides^1^		Discordant Peptides^1^	
	
	Same Sequence	Different Sequence	Same Sequence	Different Sequence
No Offsetting	44	47	5	52
Offsetting	43	27	13	24

^1^Peptides from different peptide sets with the same sequence were scored once only in a given category.

Next, the discordant peptides were compared to the corresponding positive peptides to identify the reason for non-cross-recognition. The most likely reason for non-cross-recognition was a sequence difference between the peptides. In 76 discrete epitopic regions a discordant peptide had a different sequence compared with the corresponding positive peptide, and of these 52 were in the same frame while 24 were offset in frame. Importantly, it was noted that in 18 discrete epitopic regions, discordant results were obtained between peptide sets despite sequence identity across the corresponding regions. In 13 instances there was frame offsetting between the positive and discordant peptides, which could account for the disparity in recognition. However, in 5 instances peptides of identical primary sequence were discordantly scored as positive. In each of these 5 cases the positively scored peptide only marginally exceeded the cut-off for positivity. However, it was impossible to determine if these discordantly scored peptides were a result of a false positive (peptide incorrectly scored positive) or a false negative (discordant peptide incorrectly scored negative). In summary, the majority of examples of discordant detection of CTL responses were due to sequence variation, while the position of a minimal epitope within a screening peptide inconsistently affected detection of CTL responses. Artifactual false positive or false negative detection of CTL responses was also encountered on rare occasions.

### Epitopes commonly targeted in subtype B and C are also targeted in subtype A infection

The most frequently recognized peptides (3 or more responders) were examined for the possession of common HLA alleles among the responder subjects and analyzed for known CTL epitopes. Table 3 outlines 11 commonly recognized peptides (15-mer) or peptide overlaps (11-mer) observed in the study and their probable HLA-restrictions. Of these 11 epitopes 8 have been described before [15] and are commonly recognized in both subtype B [10,11] and subtype C [25,26,31] infections. The previously described minimal epitopes are also listed in Table 3. One Nef peptide contains a previously defined HLA-B*3501 restricted epitope, or overlapping epitope, which is restricted by B*4201/02 and/or B*5301. Seven of the epitopes were detected with all peptide sets while 3 displayed variable cross-reactivity and 1 was subtype A Nef-specific. The well characterized and studied SL9 A*0201-restricted Gag p17 epitope (present in the A-Consensus and C-Isolate peptide sets) was recognized by 5 of 9 A*0201 subjects, but the variant (SLFNTIATL) was recognized by only 3 of these 5 subjects, indicating sequence dependent modulation of immunodominance and cross-reactivity for this epitope. Variable cross-reactivity was also observed for another previously characterized epitope – the Gag p24 KF11 B*5701/03 restricted epitope – was recognized by 5 of 5 B*5701/03 subjects, but the variant (KGFNPEVIPMF) was recognized by only 3 of these subjects. The B*4202 restricted Gag p24 epitope TL9 (TPQDLNMML) showed subtype specificity, in that of 4 B*4202 subjects responding to this epitope only 1 cross-reacted with the peptides containing the variant TPQDLNTML. Therefore, the M to T change in this epitope appears important for CTL recognition. Of the 4 Nef responses which could be assigned to a particular HLA-allele, only the potential A*6802 restricted epitope (AVTSSNVNHPS) showed subtype specificity. Three A*6802 subjects responded to this epitope in the A-Nef peptide set, but none recognized the complementary peptides in the C- or D-Nef peptide sets. A deletion of 2 amino acids in the A-Nef isolate sequence relative to the C- and D-Nef isolates used in the study most likely accounts for this difference in recognition. The 3 other Nef epitopes which could assigned to HLA-alleles had been defined previously and were recognized and conserved in the 3 peptide sets. Seven subjects recognized the p24 peptide FRDYVDRFFKTLRAE (amino acids 293–307; HXB2 numbering) in all of the peptide sets. This sequence maps to a highly conserved region of p24 and is identical among 3 of the peptide sets and has a single substitution of F301Y in the UG57143 (subtype D) peptide set. In the subtype C peptide set, 2 peptides (#73 and #74) have this sequence spread across their overlap, and both were recognized by all 7 subjects. Further characterization of this epitope is performed below.

**Table 3:** Summary of observed HLA associations of commonly recognized peptides and previously defined eptiopes contained within them.

Peptide set (protein)	Sequence	Number of responders	HLA allele in common	Previously defined epitope
A-Con^1^/C-Iso^2 ^(Gag)	SLYNTVATLYC	5	A*0201	SLYNTVATL
A-Iso/D-Iso (Gag)	SLFNTIATL(Y/W)C	3		
A-Con (Gag)	QSLSPRTLNAW	3	B*5703	LSPRTLNAW
A-Con/C-Iso/D-Iso (Gag)	EKAFSPEVIPMFSAL	5	B*5701/03	KAFSPEVIPMF
A-Iso (Gag)	EKGFNPEVIPMFSAL	3		
A-Con/A-Iso (Gag)	EGATPQDLNMMLNIV	4	B*4202	TPQDLNMML
C-Iso/D-Iso (Gag)	EGATPQDLNTMLNTV	1		
A-Con (Gag)	FRDYVDRFFKTLRAE	7	*C*_W_*0304	YVDRFFKTL
A-Con (Gag)	MKDCTERQANFLGKI	2	A*0101/03	ND^3^
A-Iso (Nef)	AVTSSNVNHPS	3	A*6802	ND^3^
A-Iso (Nef)	FPVRPQVPLRPMTYK	4	B*4201/02	VPLRPMTY
A-Iso (Nef)	FPVRPQVPLRPMTYK	2	B*5301	(B*3501)
A-Iso (Nef)	KKRQEILDLWVYHTQ	5	*C*_W_*0701	KRQEILDLWVY
A-Iso (Nef)	GIRYPLTFGWCFKLV	3	B*5301	YPLTFGWCY

^1^Con = Consensus peptide set

^2^Iso = Isolate peptide set

^3^ND = Not defined previously

### Characterization of an Immunodominant HLA-C_w_*0304 Restricted Gag Epitope

**Figure 6:**
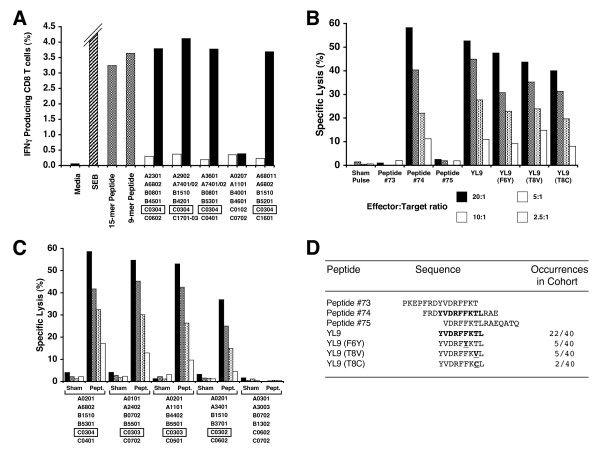
**A. **The YL9 response is *HLA-C*_W_*0304 restricted. CFC assays for IFNγ up-regulation were performed using 15-mer peptide pulsed (solid bars) or sham-pulsed (open bars), partially HLA-matched BLCL as antigen-presenting cells for PBMC. Media alone, and media containing SEB (hatched bars), served as negative and positive controls respectively. The SEB response has been truncated for clarity and the frequency of responding cells appended beside the bar. All panels were gated based upon the CD3^+^CD8^+^CD69^HIGH ^cell population in the PBMC. Only BLCL possessing the *HLA- C*_W_*0304 allele (boxed) can present the peptide. The parent 15-mer (peptide #74 from A-consensus peptide set), and the minimal 9-mer peptide (YL9) added alone to the PBMC stimulated an equivalent frequency of CD8^+ ^T cells. **B. **A CTL line generated by *in vitro *stimulation with peptide #74 from the A-Gag consensus peptide set efficiently lysed autologous BLCL pulsed with the same peptide, but not adjacent peptides #73 or #75, in a standard chromium release assay. Autologous BLCL pulsed with the predicted minimal epitope (9-mer YL9), and three variants of this epitope detected in the same cohort, were also lysed. Symbols for the effector to target ratios are denoted below the figure panel. **C. **Effector cells from the re-stimulated CTL line from above were able to lyse YL9-pulsed allogeneic BLCL, which express different alleles from the *HLA- C*_*W*_*03 *family. For each BLCL tested sham- and peptide pulsed targets are shown at a range of E:T ratios from 20:1 down to 2.5:1. The symbols for the E:T ratios are the same as used in panel 6B. The left-most BLCL on the panel are the autologous cells (*HLA-C*_W_*0304 positive), and right-most BLCL are negative for *HLA- C*_*W*_*03 *alleles. **D. **Sequences of the peptides used for the minimal epitope mapping and variant peptide testing.

**Figure 7:**
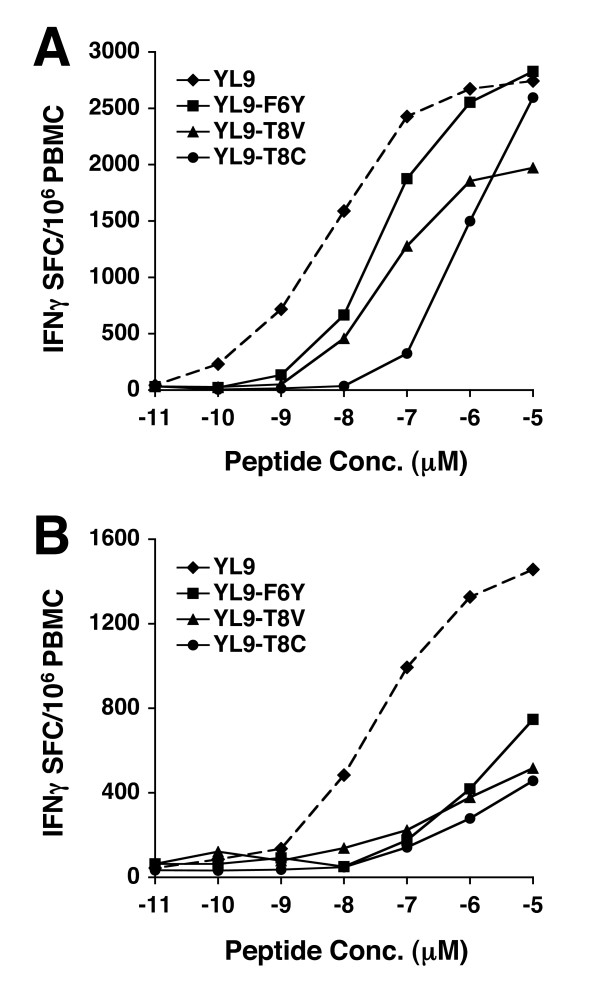
CTL response of two *HLA-C*_W_*0304 positive subjects (**A. **KNH1237; **B. **KNH1263) against YL9 peptide and three variants as measured by IFNγ ELIspot assay. Peptides were titrated through a concentration range of 10^-5 ^to 10^-11 ^M as denoted. The dashed line denotes the YL9 index peptide for clarity.

The immunodominant, cross-subtype response directed against the conserved sequence FRDYVDRFFLTLRAE (peptide #74 from the A-Gag consensus set) was further characterized. Several epitopes have been previously mapped to this region and hence it was of interest to map the minimal epitope and to determine if this response was restricted by one, or multiple, HLA alleles. All of the seven subjects responding to this epitope expressed the *HLA-C*_W_*0304 allele (or a closely related *HLA-C*_*W*_*03 *family allele). While no exact match to the defined C_*W*_0304 peptide-binding motif [34] was found in the 15-mer, an internal 9-mer (YVDRFFKTL; referred to as YL9) had the best possible match of any minimal peptide within the 15 mer peptide. Therefore, HLA restriction assays were carried out using BLCL with a partial HLA match with the test subjects and using BLCL expressing *C*_W_*0304. In addition, both the 15-mer and 9-mer peptides (YL9) were tested directly for up-regulation of IFN-γ gene expression. Representative data for one of the six responders (KNH1272) is shown in Figure 6A. The 15-mer and YL9 peptides elicit an IFN-γ response from an almost identical number of CD8^+ ^T cells (3.24% and 3.64% respectively). The only common allele expressed by the BLCL able to present the 15 mer peptide is *HLA-C*_W_*0304 demonstrating that *C*_W_*0304 is the restricting allele for this epitope. To confirm that the YL9 was the minimal epitope, CTL effector cells were generated from PBMC of a *C*_W_*0304 responder subject (KNH1241) using an *in vitro *stimulation protocol with the 15-mer peptide. Following two rounds of *in vitro *stimulation the effector cells were used in a standard chromium release assay to test for cytotoxic activity against the 15-mer peptide #74 (FRDYVDRFFLTLRAE), two flanking peptides (#73 and #75) to which no response was detected in the screening ELIspot assays, and the YL9 peptide. Figure 6B shows the efficient specific killing (effector to target ratio = 20:1) of autologous BLCL pulsed with YL9 or peptide #74 (FRDYVDRFFLTLRAE), but not peptide #73 which has the C-terminal L of YL9 truncated, nor peptide #75 which has the N-terminal Y of TL9 truncated. Therefore the minimal epitope is YL9. In addition the effector cells also demonstrated efficient killing of BLCL pulsed with variants of YL9, which were observed in the cohort. As shown in Figure 6B, BLCL pulsed with each of three variants YL9-F6Y, YL9-T8V and YL9-T8C were efficiently lysed. The ability of related alleles within the C_*W*_03 family to present the YL9 peptide was also tested. The effector cell line from KNH1241 was subjected to a further round of *in vitro *stimulation and the resultant cells used as effectors in a chromium release assay to test for cytotoxic activity against a panel of BLCL expressing different *HLA-C*_*W*_*03 *family alleles. Figure 6C shows that *C*_W_*0302 and *C*_W_*0303 can present YL9 to a *C*_W_*0304-restricted CTL line. The peptide-pulsed cells are clearly lysed at a range of E:T ratios from 20:1 to 2.5:1 compared with the sham-pulsed control cells. The autologous BLCL (*C*_W_*0304 expressing), and BLCL which do not express any *HLA-C*_*W*_*03 *alleles, were used as positive and negative controls respectively. Since the relative cross-reactivity of peptide variants can be over-estimated when using highly elevated concentrations of peptide, a titration of the response to YL9 and its variants was performed with PBMC from two YL9 responders. Figure 6D shows the sequence of the variants used and the number of occurrences of each variant in the proviral DNA of the cohort. In both subjects the response to YL9 (treated as the index peptide) was of higher avidity than that to any of the three variants (Figure 7). Of note is that the YL9-T8C variant, the weakest stimulator of all the variants, was detected in the only one of nine *C*_W_*0304 positive subjects that did not respond to the YL9 epitope. Therefore the YL9 epitope can be presented by most alleles within the *HLA-C*_*W*_*03 *family, but in spite of sequence conservation potential escape mutants may already exist.

## Discussion

While HIV-1 subtype C is the predominant circulating pure subtype in sub-saharan Africa, HIV-1 subtype A constitutes a significant proportion of the epidemic in eastern and central Africa [35-37]. Recent studies have estimated that HIV-1 subtype A constitutes >90% of the epidemic in terms of pure subtype and recombinant forms in eastern Africa, particularly in Kenya [35]. Comprehensive studies have characterized CTL responses in HIV-1 subtype B and C infections, however few studies have mapped in detail CTL cell responses in HIV-1 subtype A infections. Our results show that, as has been observed and described for subtype B and C infections, the Gag and Nef proteins are frequent targets of the CTL response of HIV-1 subtype A infections. Although the frequency of responses detected in this cohort against individual peptides was not as great as that detected in studies of subtype B infection (up to 50% of subjects responding to a single Nef peptide) [10,11], the pattern of CTL responses across the Gag and Nef proteins was remarkably similar to studies from other subtypes [10,11,26,31]. Hence, it appears likely that the N- and C-termini of p17, much of p24 and the central conserved region of Nef are immunodominant for CTL responses irrespective of the infecting subtype. Importantly, both the frequency of responding subjects, and the magnitude of the CTL responses detected against Gag and Nef using IFNγ ELIspot assay, was also consistent with results described in other studies of drug treatment naïve subjects from Africa [27,31].

Given the disparity of the different peptide sets used (derived from subtype A consensus, and subtype A, C and D isolates) and the fact that the subtype A derived peptide sets were much more similar on average to the viral sequences obtained from the studied cohort, it was surprising to observe that both the magnitude and pattern of recognition of the peptide sets was so similar for both Gag and Nef. CTL recognition is critically dependent upon peptide binding to MHC molecules and T cell receptor interactions with the peptide-MHC complex [38]; hence it was reasonable to expect that peptides from non-subtype A origin should be recognized to a lesser extent than the subtype A derived peptides. However, the results presented here demonstrate that viral sequence variability, and by extension the infecting subtype, does not predict the degree of subtype-specific responsiveness of CTL. The subtype A based peptide sets only marginally improved our ability to detect CTL responses, with no single peptide set capable of detecting all CTL responses. These observations are not without precedent, as we have demonstrated equivalent Gag, Env and Nef multiple-subtype recognition by CD8 T cells using a recombinant vaccinia virus based expression system producing full-length gene products in a similar cohort of Kenyan subjects [39]. In addition, in studies on individuals from Uganda, where subtype D and subtype A predominate in the HIV-1 epidemic, the lack of subtype-specificity and predictive capacity of the infecting subtype has also been described [27]. A simplistic interpretation of these results is that broadly cross-reactive CTL are generated by any subtype of infection and these responses may be predictive of the ability of a single isolate, or consensus sequence, based vaccine to generate broadly cross-protective responses. An important caveat associated with the experimental approach used in these studies need to be considered before such a profound conclusion can be reached with confidence. The concentration of 15-mer peptides used in these studies is very high – usually in the low micro-molar range, whereas minimal peptides have been shown to have half-maximal CTL stimulatory capacity in the low nano-molar range to high pico-molar range [40,41]. The excessive amounts of 15-mer peptide used in the screening assays may stimulate partially cross-reactive CTL, which might not recognize endogeneously processed and presented antigen on a virus infected cell. Titration of the *HLA-C*_W_*0304 restricted YL9 epitope variants performed in this study showed that even though cross-reactivity of the F6Y variant was detected for all responding subjects (8/9) in the screening and confirmation assays, CTL have a much lower avidity for the F6Y variant than the wild-type. Hence, true CTL cross-reactivity needs a more stringent definition – one that takes into account avidity for variant peptides, and translation of avidity measurements into biological relevance. This effect may be even more pronounced on HIV-1 infected cells where surface expression of HLA class I A- and B-alleles can be down-regulated by the Nef protein [42,43].

A further caveat associated with the use of 15-mer peptides is that minimal CTL epitopes – generally 9–10 amino acids in length – are embedded in the 15-mer peptides. Several recent studies have demonstrated that the relative position (or offset) of a minimal epitope within a 15-mer peptide can adversely affect the magnitude of the CTL response measured in response to a given 15-mer peptide [21,23]. While the precise mechanism behind the "epitope-offsetting" effect has not been elucidated it is assumed that amino acid residues flanking the minimal epitope may impair the ability of the 15-mer to be bound by MHC, and/or processed, prior to CTL recognition. As shown in the present study the epitope-offsetting effect can lead to artifactual false-negatives when comparing different sets of 15-mer OLPs. Hence, the high concentrations of 15-mer peptides used in screening assays may contribute to an over-estimation of cross-reactivity, and the offsetting of variant epitopes in different 15-mer peptide sets can lead to an under-estimation of cross-reactivity. Despite the potential to confound interpretation of our results, the epitope-offsetting effect was the exception rather than the rule, and as such we believe our overall interpretation of the results to be accurate. In support of this are prior studies, which have shown that multi-subtype reactivity can be readily detected using recombinant vaccinia viruses to deliver Gag, Nef and Env proteins into the cytoplasm – thereby enabling *de novo *antigen-processing and presentation to occur – avoiding concentration-dependent issues with peptide studies [28,39,44].

Reagent selection is a critical determinant for maximizing detection of CTL specific for different epitope variants of HIV-1. While the use of reagents that match the autologous virus sequence in a given host would be the ideal reagent of choice, practical limitations preclude this approach for large-scale cross-sectional screening studies. Consensus and ancestral sequence based peptide sets have been proposed as a practical solution for maximizing CTL epitope detection. However, as the data presented here shows, a consensus sequence representing a single subtype cannot fully capture the breadth of the CTL response. Utilization of multiple peptide sets clearly increases the breadth of detection of HIV-1-specific CTL, most likely because of the presence of different variants of many epitopes among the peptide sets. Using peptides representing different variants of epitopes – either in OLP sets or as minimal peptide epitopes – therefore gives a better account of the true CTL breadth, especially in locations where multiple subtypes co-circulate. The importance of revealing the true breadth of the CTL response in HIV-1 infection has been highlighted in the recent study of Frahm et al. [45], which showed that targeting of subdominant CTL epitopes was associated with better control of viral load. If this result proves to be generalizable, then CTL-based vaccine and immunotherapeutic strategies will need to maximize the breadth of the induced CTL response in order to be efficacious. This further highlights the importance of reagent selection for measuring CTL magnitude and breadth and for interpreting cross-reactivity in the setting of HIV-1 infection and vaccine trials.

While several HLA-C restricted epitopes from HIV-1 have been defined [15], this study presents, to our knowledge, the first evidence of HLA-C restricted immunodominance at the population level. Interestingly, the minimal 9-mer (YL9) defined here (and in reference [25]) with a *HLA-C*_W_*0304 allelic restriction, has been defined with HLA-B*1510 [31] and HLA-A*0207 [44] restrictions in other populations. However, the B*1510 restriction analysis of this peptide defined by Novitsky *et al *[31] did not completely rule out the possibility that HLA-C may have been the restricting locus because of the tight linkage disequilibrium of the HLA-B*1510 allele with *C*_*W*_*03 *alleles. Given that YL9 is recognized in the context of most members of the *HLA-C*_*W*_*03 *allele family, and at least two other HLA alleles, and is derived from a highly conserved region of the p24 protein, this peptide warrants inclusion in minimal epitope based vaccine strategies. It should be noted that the most frequently observed peptide response and HLA combination that we noted for the Nef protein was also a HLA-C allele: *HLA-C*_W_*0701 with peptide sequence KKRQEILDLWVYHTQ (defined as KY11 associated with *C*_W_*0701 allele possession in reference [25]). HLA-C alleles may offer an attractive alternative to the more commonly proposed HLA-A and -B loci for minimal epitope targeted vaccine strategies because they are refractory to down-regulation by the Nef protein [42,43].

## Conclusion

In conclusion, we have shown that in subtype A infections the pattern of immunodominance and epitope clustering observed within the Gag and Nef proteins of HIV-1 is similar to that seen in subtype B and C infections. Importantly, similar epitope clustering patterns were observed using either a subtype matched consensus-based peptide set, or isolate-based peptide sets from heterologous subtypes. In agreement with previous studies, knowledge of the predominant infecting subtype in a given population does not necessarily predict the subtype-specificity of the CTL response. An extension of this observation was the finding that no single screening sequence, even a subtype matched consensus sequence, can fully capture the true breadth of the CTL response. Future studies aimed at capturing the true breadth and cross-reactivity of HIV-1-specific CTL responses in multiple-subtype endemic regions will require: (1) monitoring the viral quasi-species longitudinally to give a sense of what sequences may be driving a measured CTL response; (2) high resolution HLA-typing data from the subjects studied, (3) titration studies with synthetic peptides representing minimal epitopes of multiple variants or peptides based upon the autologous viral sequence and; (4) an understanding of how closely the avidity of a CTL for a variant peptide needs to match that for the index peptide to be considered truly cross-reactive. Finally, vaccine trials offer the best setting for testing the true extent CTL cross-reactivity, since this is the only circumstance in which the exact sequence that primes a CTL response is known with certainty.

## Methods

### Study subjects

Anonymously donated HIV-1 positive blood units were collected between 1999 and 2000 from Kericho District Hospital (Kericho), Rift Valley Provincial Hospital (Nakuru) and Kenyatta National Hospital (Nairobi), all in southern Kenya, under a study approved by both Kenyan and U.S. based Institutional Review Boards. Names, personal information and medical conditions of the subjects were not available, but all subjects were antiretroviral treatment naïve. Blood units were identified only by an alphanumeric code and because of the nature of collection, CD4 counts and viral load data were not available. HIV-1 positivity was assessed by Serostrip (Saliva Diagnostic Systems, Medford NY, USA) and confirmed by ELISA (Organon Teknika/BioMerieux, Inc., Marcy l'Etiole, France).

### PCR, cloning and sequencing of HIV-1 *gag *and *nef*

Peripheral blood mononuclear cell DNA was extracted using QIAamp blood extraction kit (QIAGEN, Valencia, CA). A nested PCR was employed to retrieve *gag *and *nef *region. The PCR mixtures and cycling conditions were described previously [46]. For *gag *region, outer primers used to amplify *gag *(HXB2 positions 796 to 2381) were MSF12B (5'-AAATCTCTAGCAGTGGCGCCCGAACAG -3') and BJPOL3 (5'- GTTGACAGGTGTAGGTCCTAC-3'). Then either using F2NST (5'-GCGGAGGCTAGAAGGAGAGAGATGG -3') and SP3AS (5'-CCTCCAATTCCCCCTATCATTTTTGG-3') as the inner primers and directly sequenced the amplicon obtained, or performing the second-round PCR using two inner primer pairs, F2NST/SP3AS and GAG763 (5'-TGACTAGCGGAGGCTAGAAGGAGAGA-3')/JL80 (5'-TAATACTGTATCATCTGCTCCTGT-3'). The PCR products were pooled, purified and cloned into the pCR2.1-TOPO vector using a Topo TA cloning kit (Invitrogen Corp., Carlsbad, CA). *Nef *region from HXB2 positions 8797 to 9417 was amplified. Outer primers were JL106 (5'-TTCAGCTACCACCGCTTGCGAGACT-3') and UNINEF 7 (5'-GCACTCAAGGCAAGCTTTATTGAGGCTT-3'). The second-round PCR was done using two different inner primer pairs. The first pair was UVNEF1 (5'-GGGGTCGGGAACTGAAAATTAGTGC-3') and TATANEF (5'- GCAGCTGCTTATATGCAGGATCTGAGGG-3'). The second pair was UVNEF2 (5'-AGACAGGGCTTTGAAAGGGCTTT-3') and UNINEF7. The two PCR products obtained were pooled, purified and subjected to sequencing. Plasmid DNA or PCR product was directly sequenced using Big Dye terminator reaction kits and an Applied Biosystems 3100 capillary sequencer (Applies Biosystems Inc., Foster City, CA). The obtained sequences were aligned with reference sequences of the relevant subtypes and circulating recombinant forms (CRF). Phylogenetic analysis was performed to subtype designate the sequences of interest using SEQBOOT, DNADIST (Kimura 2-parameter, transition/transversion ratio = 2.0), NEIGHBOR, and CONSENSE modules of the PHYLIP Package. Deduced *gag *and *nef *protein were generated from the nucleotide sequences and aligned. Pair-wised distance matrix of proteins was calculated using maximum likelihood estimates based on the Dayhoff PAM matrix.

### PCR-based HLA class I Typing

HLA typing was performed on all subjects at the University of Alabama (Birmingham, AL), using DNA extracted from either PBMC or autologous B lymphoblastoid cell lines (BLCL). Briefly, high molecular weight genomic DNA was extracted from immortalized B cells or PBMC using the QIAamp Blood Kit and protocols recommended by the manufacturer (Qiagen Inc., Valencia, California, USA). The 4-digit alleles at the HLA-A and -B loci were resolved by automated reference-strand conformation analyses (RSCA) of PCR amplicons corresponding to exon 2, intron 2, and exon 3 sequences (Pel-Freez Clinical Systems, Brown Deer, Wisconsin, USA). The 2-digit HLA-C specificities were defined separately by PCR with sequence-specific primers (SSP) (Pel-Freez Clinical Systems, Brown Deer, Wisconsin, USA).

### Synthetic peptides

PBMC were screened for HIV-specific CTL responses by stimulation with OLPs representing Gag and Nef from isolates of subtypes A, C and D. OLPs consisted of 15-mer peptides overlapping by 11 amino acids covering the entire Gag and Nef protein sequence of isolates 90CF402 (subtype A-*gag*, ACC# AAB38823), DU422 (subtype C-*gag*, ACC# CAD62240) 98UG57143 (subtype D-*gag*, ACC# AF484514), 92UG037 (subtype A-*nef*, ACC# AAC97549), DU151 (subtype C-*nef*, ACC# AAL05314) and 94UG114 (subtype D-*nef*, ACC# AAC97574). Peptides were synthesized using FMOC chemistry and standard solid-phase techniques with free amino termini. All peptides were >80% pure as determined by HPLC, mass spectroscopy, amino acid analysis and N-terminal sequencing. The A-Gag peptide set was synthesized at the Henry M Jackson Foundation and by Anaspec Inc. (San Jose, CA), while the C-Nef peptide set was a kind gift from Dr. Clive Gray (National institute for Communicable Diseases, Johannesburg, South Africa). All other peptide sets were synthesized at the Natural and Medical Sciences Institute (University of Tuebingen, Germany). Synthetic peptide epitopes of 9 amino acids in length with free amino termini were synthesized using FMOC chemistry and standard solid-phase techniques (Excel automated synthesizer; Waters, Milford MA). Syntheses were carried out in-house (Henry M. Jackson Foundation). All peptides were >80% pure as determined by HPLC, and verified for correct sequence by mass spectroscopy, amino acid analysis and N-terminal sequencing. The peptide pool matrices were of the following formats: 11 linear pools of 11 or 12 peptides and 11 pools of every 11^th ^peptide for the Gag peptide sets; 7 linear pools of 7 or 8 peptides and 7 pools of every 7^th ^peptide for the Nef peptide sets. Therefore, less than two whole 96-well ELIspot plates were required to screen all 7 peptides sets for each individual. See Additional file 1: Peptide Sequences for the list and sequence of all synthetic peptides used in the study.

### Enzyme-linked immunospot (ELIspot) assay

All assays were performed using RPMI-1640 medium containing 10% normal human serum, 100 U/ml penicillin and 100 μg/ml streptomycin (complete medium; CM). Cryopreserved PBMC were recovered from liquid nitrogen, thawed rapidly, washed twice with CM and incubated at 2–5 × 10^6 ^cells/ml in CM for 14–16 hours. After the overnight rest, the PBMC were enumerated prior to addition to the ELIspot assay plates at 1–2 × 10^5 ^viable cells per well. Ninety-six well ELIspot plates (Multiscreen^®^-IP plates MAIP type plates, Millipore, MA) were prepared by pre-wetting with 50 μl of 70% ethanol (in water) per well and incubating for 5 minutes at room temperature followed by washing six times with PBS. Plates were coated with mouse anti-human IFNγ monoclonal antibody (1-D1K, Mabtech AB, Sweden) at 5 μg/ml in 100 μl PBS overnight at 4°C. Plates were washed six times with PBS and blocked with 100 μl of complete medium for one hour at 37°C. As a positive control for functional integrity of the cells, staphylococcal entertoxin-B (SEB) was added to the wells at 5 μg/ml final concentration and cells were incubated with CM only as a negative control. Peptides were used at a final concentration of 2 μg/ml and all ELIspot tests and controls were performed in triplicate wells. Plates were incubated for 20–24 hours at 37°C (5% CO_2_), washed six times with PBS/0.05% Tween 20 buffer and incubated for two hours with biotinylated mouse anti-human IFNγ monoclonal antibody (7B6-1-biotin, Mabtech AB, Sweden) at 2 μg/ml in PBS/0.05% Tween 20 buffer. ELIspot development consisted of a one hour incubation with an avidin horseradish peroxidase complex (Vectastain^® ^ABC kit, Vector Labs, CA) in PBS/0.05% Tween 20 buffer followed by washing six times with PBS, and incubation with peroxidase substrate AEC for five minutes (AEC substrate Kit, Vector Labs, CA). ELIspot plates were examined under a stereomicroscope and spots were evaluated with an Automated Elispot Reader System using KS 4.3 software (Carl Zeiss, Thornwood, NY) by an independent scientist in a blinded fashion (Henry M. Jackson Foundation, Rockville, MD). Positive IFNγ spot forming units (SFU) representing single cells were counted and expressed as SFU per one million input PBMC. Assays were considered valid if responses of greater than 500 SFU per 1 million PBMC were detected with SEB. In the screening assays the seven peptide sets were tested in a matrix format consisting of 11 pools of 11 or 12 peptides for the Gag OLP sets and 7 pools of 7 or 8 peptides for the Nef OLP sets. After matrix deconvolution most of the individual peptides were screened in triplicate to confirm the response. Positive responses were defined as those where the mean of the test wells exceeded the 99% confidence interval of replicates of six negative control wells.

### CTL effector cell generation

Effector cells were prepared by *in vitro *stimulation of thawed cryopreserved PBMC. Peptide pulsed (10 μg/ml overnight), irradiated (10000 rad) autologous BLCL (2 × 10^6 ^cells) were washed three times with CM and then co-cultured with 10 – 20 × 10^6 ^PBMC in 10 ml CM supplemented with 5 ng/ml rhIL-7 (R&D Systems, Minneapolis, MN) for 4 days. 5 ng/ml rhIL-2 (R&D Systems, Minneapolis, MN) was then added to the co-cultures, and the cultures were maintained and split with fresh CM and rhIL-2 for up to 24 days. Effector cell lines were then maintained by re-stimulation every 7–14 days with irradiated, peptide pulsed, autologous BLCL and 5 ng/ml rhIL-2 in CM.

### Chromium release assay for cytotoxic activity

CTL effector cells prepared as above were screened for cytotoxic activity against peptide pulsed autologous or partially HLA matched BLCL. BLCL (1 × 10^6^) were incubated overnight with 10 μg/ml of relevant peptide in the presence of 100 μCi of ^51^Cr-labelled sodium chromate (NEN, Boston, MA) and then washed three times with RPMI-1640 to remove excess ^51^Cr-sodium chromate and peptide. Non-peptide pulsed BLCL served as the negative control for cytotoxic activity. CTL activity was titrated at several effector to target (E:T) ratios and expressed as percentage of maximal specific lysis of 2500 ^51^Cr-labelled target cells per well. Maximum release was determined by lysis of ^51^Cr-labelled target cells with 5% SDS. Percent specific cytotoxicity was calculated using the formula: 100 × [(test release - spontaneous release)/(maximum release - spontaneous release)]. Assays were considered valid if spontaneous release was <30%. All assay tests were performed in triplicate and a specific activity of >10% was considered to be positive.

### Cytokine flow cytometry (CFC) assay

Cryopreserved PBMC were thawed rapidly, washed twice with CM and either used immediately, or incubated at 2–5 × 10^6 ^cells/ml in CM for 14–16 hours. PBMC were added at 0.5 to 1.0 × 10^6 ^cells per well into 96 well polypropylene tissue culture trays and stimulated either directly with SEB, or with autologous or allogeneic peptide-pulsed BLCL. BLCL were pulsed overnight with relevent peptide (10 μg/ml), washed five times after the overnight incubation, and distributed at 1 × 10^5 ^cells per well (ratio 10:1, PBMC:BLCL). As a positive control for functional integrity of the cryopreserved cells, SEB was added to a single well at 5 μg/ml final concentration. The assays were incubated for 6 hours at 37°C (5% CO_2_) in the presence of the protein transport inhibitor Brefeldin A (10 μg/ml; Sigma, St.Louis, MO), and were then terminated by transfer to 4°C overnight. Cells were stained for surface markers and intracellular IFNγ expression (CFC assay) the following day. Co-cultured PBMC (prepared above) were washed once with flow buffer (DPBS/0.1% BSA/0.1% sodium azide) and incubated in the 96-well tissue culture tray wells for 10 minutes in 200 μl flow buffer (containing 1 mM EDTA) at room temperature (same volume, temperature and base buffer used for all subsequent washings and incubations). Cells were washed once, fixed in 2% formaldehyde for 30 min and washed again. Fixed cells were permeabilized with 0.5% saponin (Sigma, St. Louis MO.) for 30 min, washed and resuspended in 0.5% saponin containing the following monoclonal antibodies: FITC-conjugated anti-interferon-γ (clone 25723.11); PE-conjugated anti-CD69 (clone L78); PerCP-Cy5.5-conjugated anti-CD8 (clone SK1); and APC-conjugated anti-CD3 (clone SK7)(BD Biosciences, San Jose, CA). After 30 min incubation, cells were washed three times with flow buffer and finally re-suspended in 200 μl of flow buffer. Stained cells were stored at 4°C and analyzed by flow cytometry within 24 hours. Data acquisition was performed on a FACScalibur flow cytometer (Becton Dickinson, San Jose, CA). Initial gating was performed using a total lymphocyte gate based on forward and side scatter characteristics and acquisition of 50, 000 – 200, 000 cells within this gate. Color compensation was performed using similarly prepared cells from an HIV-1 sero-negative donor and staining singly labeled cells with anti-CD3 labeled with FITC, PE, PerCP-Cy5.5 and APC fluorochromes (BD Biosciences). Data sets were analyzed using FlowJo software (version 4) (TreeStar, Cupertino, CA). Test wells were considered positive if at least a two-fold increase in gated positive events was detected compared with the appropriate negative control. The mean background IFNγ positive CD8 T cells was 0.026% and 0.81% for the media only controls and sham-pulsed BLCL respectively.

## Authors' contributions

JRC and JHC designed the study and participated in statistical analysis and final data interpretation. JRC and UV performed the experiments and UV, ST and FEM analyzed the sequence data. JRC wrote the manuscript and JHC and FEM edited the final versions. DLB and CJM participated in cohort development and sample acquisition from the field sites. All authors have read and approved the final version of the manuscript.

## Supplementary Material

Additional File 1 Description of data: Listed are the amino acid sequences of 638 synthetic peptides that were used in the study. The GenBank accession number for the nucleotide sequence from which the protein sequence was translated is denoted. Click here for file
